# Proteinase-Mediated Macrophage Signaling in Psoriatic Arthritis

**DOI:** 10.3389/fimmu.2020.629726

**Published:** 2021-03-08

**Authors:** Fatima Abji, Mozhgan Rasti, Alejandro Gómez-Aristizábal, Carla Muytjens, Mahmoud Saifeddine, Koichiro Mihara, Majid Motahhari, Rajiv Gandhi, Sowmya Viswanathan, Morley D. Hollenberg, Katerina Oikonomopoulou, Vinod Chandran

**Affiliations:** ^1^ Schroeder Arthritis Institute, Krembil Research Institute, University Health Network, Toronto, ON, Canada; ^2^ Department of Physiology & Pharmacology, University of Calgary Cumming School of Medicine, Calgary, AB, Canada; ^3^ Division of Orthopaedic Surgery, Department of Surgery, Toronto Western Hospital, Toronto, ON, Canada; ^4^ Institute of Biomedical Engineering, University of Toronto, Toronto, ON, Canada; ^5^ Division of Hematology, Department of Medicine, University of Toronto, Toronto, ON, Canada; ^6^ Department of Medicine, University of Calgary Cumming School of Medicine, Calgary, AB, Canada; ^7^ Division of Rheumatology, Department of Medicine, University of Toronto, Toronto, ON, Canada; ^8^ Institute of Medical Science, University of Toronto, Toronto, ON, Canada; ^9^ Department of Laboratory Medicine and Pathobiology, University of Toronto, Toronto, ON, Canada; ^10^ Department of Medicine, Memorial University of Newfoundland, St. John’s, NL, Canada

**Keywords:** serine proteinase, spondyloarthritis, monocyte, macrophage, synovial fluid, PAR2, tryptase-6

## Abstract

**Objective:**

Multiple proteinases are present in the synovial fluid (SF) of an arthritic joint. We aimed to identify inflammatory cell populations present in psoriatic arthritis (PsA) SF compared to osteoarthritis (OA) and rheumatoid arthritis (RA), identify their proteinase-activated receptor 2 (PAR2) signaling function and characterize potentially active SF serine proteinases that may be PAR2 activators.

**Methods:**

Flow cytometry was used to characterize SF cells from PsA, RA, OA patients; PsA SF cells were further characterized by single cell 3’-RNA-sequencing. Active serine proteinases were identified through cleavage of fluorogenic trypsin- and chymotrypsin-like substrates, activity-based probe analysis and proteomics. Fluo-4 AM was used to monitor intracellular calcium cell signaling. Cytokine expression was evaluated using a multiplex Luminex panel.

**Results:**

PsA SF cells were dominated by monocytes/macrophages, which consisted of three populations representing classical, non-classical and intermediate cells. The classical monocytes/macrophages were reduced in PsA compared to OA/RA, whilst the intermediate population was increased. PAR2 was elevated in OA vs. PsA/RA SF monocytes/macrophages, particularly in the intermediate population. PAR2 expression and signaling in primary PsA monocytes/macrophages significantly impacted the production of monocyte chemoattractant protein-1 (MCP-1). Trypsin-like serine proteinase activity was elevated in PsA and RA SF compared to OA, while chymotrypsin-like activity was elevated in RA compared to PsA. Tryptase-6 was identified as an active serine proteinase in SF that could trigger calcium signaling partially *via* PAR2.

**Conclusion:**

PAR2 and its activating proteinases, including tryptase-6, can be important mediators of inflammation in PsA. Components within this proteinase-receptor axis may represent novel therapeutic targets.

## Introduction

Inflammation is central to the pathogenesis of arthritis. In psoriatic arthritis (PsA) the cells involved in bone formation (osteoblasts) and resorption (osteoclasts), synoviocytes and immune cells (such as macrophages, neutrophils, mast cells, T cells and B cells) are sources of inflammatory components within the synovial space (1, 2). A greater understanding of the role of cytokines such as TNFα and IL-17 has led to the development of effective therapies. For example, targeting the IL-23–IL-17 pathway is effective in the management of PsA (3). However, almost 40% of patients fail to show a clinically meaningful response with these agents (4–6) and pain and inflammation persist, particularly when these therapies are not introduced early in the disease course. Thus, it is critical to identify the underlying pathogenic mechanisms that lead to inflammation, joint damage, and persistent pain. This insight will generate new targets for therapies that can halt or reverse progressive joint damage, provide pain relief and improved the quality of life of patients with PsA.

Proteolytic activity is crucial to the maintenance of cartilage and bone integrity in the joint. Excessive activity of proteinases (7), including matrix metalloproteinases (MMPs) (8, 9) and cysteine proteinases, such as cathepsins (10), are involved in bone resorption and the degradation of proteins in the cartilage, tendons and bone extracellular matrix (ECM) (8, 11, 12). Elevated levels of MMPs (in particular MMP-3) have been detected in blood, synovial tissue and synovial fluid (SF) samples obtained from patients with spondyloarthritis (SpA), including PsA (13–15). Serine proteinases such as those of the coagulation and fibrinolysis cascades and those involved in the complement cascade are another group of enzymes of potential importance in arthritis (16–21). Our analysis of SF from patients with PsA has revealed kallikrein-related serine peptidases (22).

Proteinase-activated receptors (PARs) include a family of four G-protein-coupled receptors with pro-inflammatory functions (23, 24). Serine proteinases, cysteine proteinases and metalloproteinases can activate three of these receptors (PAR1, PAR2 and PAR4), unmasking a self-activating ligand. Activation of PARs is linked to joint inflammation, damage and pain in both mouse models and human arthritides (25–36), but PAR expression and activity in PsA has not been reported.

The upstream activators of PARs within the human joint have not been elucidated. Serine proteinases regulate PAR function in other settings such as colitis and skin inflammation and inhibition of these proteinases in murine models of arthritis has pointed to their role as potential PAR activators in human arthritides (23, 37, 38). Most studies on PARs in arthritis have relied on rodent models of inflammation and studies in patient cohorts are lacking. The aim of this study was to characterize the immune effector cell populations in SF of human PsA patients, their PAR expression, and to identify proteinase mediators that might influence PAR signaling and function. For the purpose of this study, we focused on PAR2, known to be implicated in inflammation and pain (7) and its major activators, serine proteinases. SF cells from rheumatoid arthritis (RA) patients, a well-known inflammatory arthritis, and osteoarthritis (OA) patients, a commonly used control characterized by less inflammation, were used as reference. First, we characterized the cell populations present in the SF of individuals with PsA compared to OA and RA and their expression of PAR2, with a focus on monocyte/macrophages, known to be a source of several proteinases in inflammatory arthritis (39). Next, we identified serine proteinases in PsA SF and their potential impact on signaling *via* PAR2. Finally, we sought to investigate a role for PAR2 in regulating monocyte/macrophage function in PsA.

## Materials and Methods

### Study Subjects

PsA patients (n=56) with available SF samples from the knee joint were selected from a cohort of patients followed prospectively at the University of Toronto PsA clinic. All PsA patients had psoriasis confirmed by a dermatologist and satisfied the ClASsification for Psoriatic ARthritis (CASPAR) study group criteria (40). Additional SF samples from the knee joint were obtained from patients with RA (n=22) and OA (n=30) for comparison. SF was obtained during routine joint aspirations (PsA, RA) or arthroscopy (OA). Patients with OA had a grade II-IV knee OA by the Kellgren-Lawrence classification system (41). This study was approved by the University Health Network Research Ethics Board and was conducted according to principles of the Declaration of Helsinki. All participants provided written informed consent.

### Flow Cytometry and Single Cell 3’-RNA-Sequencing

Cells from SF samples were separated by centrifugation and cells from OA patients were labeled immediately due to the low numbers of cells present. For PsA and RA patients, cells were stored at −80°C in RPMI medium supplemented with 20% FBS and 10% DMSO until ready for analysis. Single cell 3’-RNA-sequencing was performed on samples from patients with PsA (n=3) using the 10X Genomics Chromium platform (42, 43). A total of 1000 cells were captured for each sample and sequencing was performed to a depth of 60,000 reads per cell on the Illumina NextSeq 500. The quality control metrics were obtained using RNA-SeQC (v1.1.7). The raw FASTQ data files were aligned to the human genome (GRCh38) using the STAR aligner (STAR v2.5.2b). Gene-barcode matrices were obtained using the CELLRANGER (v3.0.2) pipeline. These were further loaded into R (v3.6.1) for the final graphical output of results and statistical analysis.

Flow cytometry was used to characterize cell subtypes present in SF from PsA, RA and OA patients (n=10 in each group) and to identify PAR2- and CCR2-expressing cell populations. Cells (when frozen) were thawed rapidly at 37°C and filtered through a 35 µm cell strainer and labeled using the following markers or appropriate isotype controls: CD45-pacific blue (clone 2D1), CD14-PE-Cy7 (clone 63D3), CD16-BV510 (clone 3GB), HLA-DR-PerCP-Cy5.5 (clone L243), CCR2-APC-Cy7 (clone KO36C2) and PAR2-PE (Santa Cruz Biotechnology, Dallas, TX, USA, clone SAM11). Fluorophore-conjugated antibodies were purchased from BioLegend (San Diego, CA, USA) or as indicated above. Data were acquired using a BD FACS Canto II and analysis was performed using FlowJo (v10). Gating was performed based on a previously described method (44).

### Fluorogenic Proteinase Activity Assay

Proteinase activity was measured in SF samples by measuring cleavage of fluorogenic substrates for trypsin-like [Boc-Val-Pro-Arg- Aminomethylcoumarin (VPR-AMC), I-1120.0050, and H-D-Val-Leu-Lys- Aminomethylcoumarin (VLK-AMC), 4008009.0050, Bachem Laboratories, Bubendorf, Switzerland] and chymotrypsin-like [Suc-Ala-Ala-Pro-Phe- Aminomethylcoumarin (AAPF-AMC), Bachem Laboratories, 4012873.005] proteinases. Assay buffer [phosphate-buffered saline (PBS), pH=7.5] was combined with substrate (1 mM for VPR-AMC and VLK-AMC; 2 mM for AAPF-AMC) and 10 µl of samples (SF or enzyme) in a total reaction volume of 100 µl in 96-well plates. Fluorescence activity was measured on a FLUOstar Omega plate reader at excitation of 380 nm and emission of 460 nm (BMG Labtech, Ortenberg, Germany). Fluorescence release due to substrate cleavage was monitored for 20 min at 37°C to generate a kinetic curve [fluorescence units (FU)/min]. Trypsin-like or chymotrypsin-like proteinase activity was determined relative to a standard curve of fluorescence release by different concentrations of active trypsin (Millipore Sigma, Burlington, MA, USA, T0303) or chymotrypsin (Millipore Sigma, C3142). All samples were run in duplicate and normalized to total protein levels (in mg). The Pierce BCA Protein Assay kit was used to measure total protein levels (Thermo Fisher Scientific, Waltham, MA, USA, 23227).

### In-Gel Trypsin Digestion and Identification of Proteinases by Mass Spectrometry

SF samples from PsA, RA and OA patients (n=2 in each group) were run on precast PAGEr Tris-Glycine Gold 4–20% gels under reducing conditions to identify potential serine proteinases based on size (Lonza, Basel, Switzerland, 59522). Proteins were stained using Coomassie Brilliant Blue R-250 Staining Solution (Bio-Rad Laboratories, Hercules CA, USA, 1610436) and individual bands were excised and digested based on a previously described protocol (45). Peptides were analysed on the Q Exactive Plus Mass Spectrometer (Thermo Fisher Scientific). Proteins were identified using MaxQuant (46), with a false discovery rate (FDR) of 0.01 as the filtering criteria at both the protein and peptide level for identification. Differences between groups were determined using the Perseus software (47). This procedure was followed by the removal of potential protein contaminants (e.g. keratin) (48) and serine proteinases were further screened based on biological function for further investigation.

### Visualization of Active Serine Proteinases in Synovial Fluid and Western Blot Analysis Following Covalent Labelling With a Biotinylated Activity-Based Probe Reagent

SF samples from PsA, RA and OA patients (n=2 per group) were reacted with a biotin-tagged serine proteinase-targeted activity-based probe, biotin-Pro-Lys-diphenylphosphonate (Bio-PK-DPP4) (49), based on a previously described protocol (50). Five microliter of SF was incubated in the presence or absence of 4 µg/ml of soybean trypsin inhibitor (STI; Millipore Sigma, T9128). Proteins were denatured at 95°C and stored at −20°C until ready for visualization by western blot analysis. Additional SF samples without reaction with the activity-based probe were prepared for analysis on a parallel western blot for detection of a serine proteinase (tryptase-6) that had been found previously to be present in the samples using in-gell trypsin digestion and mass spectrometric analysis.

The activity-based probe-labeled SF sample was diluted 1:5 using Laemmli sample buffer prior to electrophoretic analysis using a 4–15% Mini-PROTEAN TGX Precast Protein Gel under reducing conditions (Bio-Rad Laboratories, 4561086). In parallel, 2.5 µl of PsA, RA and OA SF from the same patients (n=2 each) for whom activity-based probe analyses were done, were analysed on the same gel with 5 µl of the precision plus protein standard (Bio-Rad Laboratories, 1610374). The gel was then transferred to an Immun-Blot PVDF membrane (Bio-Rad Laboratories, 1620177) and cut for separate probing to detect the activity-based probe labelled enzymes and tryptase-6. Blots to detect the biotin-tagged activity-based probe were blocked overnight at 4°C in 4% casein in Tris-buffered saline containing 0.1% Tween 20 (TBS-T), washed, and incubated for 1 h in streptavidin-HRP (Jackson ImmunoResearch, West Grove, PA, USA, 016-030-084) diluted 1:10,000 in 1% casein-TBS-T. After extensive washing, the activity-based probe-proteinase complexes were visualized by addition of ECL prime western blotting reagent (GE Healthcare, Chicago, IL, USA, 45-002-401) using the ChemiDoc MP imaging system (Bio-Rad Laboratories). Blots for tryptase-6 were blocked for 1 h at room temperature in 4% casein-TBS-T. This blocking was followed by overnight incubation at 4°C in rabbit anti-tryptase-6 antibody (Aviva Systems Biology, San Diego, CA, USA, OAAF06983) diluted 1:1,000 in 1% casein-TBS-T. The blot was then washed and incubated in goat anti-rabbit HRP secondary antibody (Thermo Fisher Scientific, 31462) diluted 1:10,000 in 1% casein-TBS-T. Blots were washed and visualized as described above.

### Tryptase-6 ELISA Quantification, Pull-Down Activity Measurement and Antibody Affinity Chromatography Isolation

Total tryptase-6 levels were measured in SF from PsA, RA and OA individuals (n=10 per group) using a commercially available ELISA (Aviva Systems Biology, OKCD02115). Samples were diluted 1:10 using the standard diluent provided with the kit.

Active tryptase-6 was detected in the SF samples using a pull-down assay adapted from a previously described protocol, whereby enzyme captured by plate-immobilized antibody retains its measurable enzymatic activity to cleave substrate in the supernatant (51). Trypsin-like serine proteinase activity was measured by cleavage of fluorogenic substrates (0.25 mM VPR-AMC or 0.5 mM VLK-AMC) using a FLUOstar Omega plate reader (BMG Labtech) set at 380 nm for excitation and 460 nm for emission at 37°C over a 60 min time interval. The slopes of the resulting kinetic plots (FU/min) were normalized to either total protein (mg) or total tryptase-6 (ng).

Total tryptase-6 was isolated from PsA SF samples (n=2) for treatment of cells in the assays described below using the catch and release reversible immunoprecipitation (IP) antibody affinity chromatography system, according to the manufacturer’s instructions (Millipore Sigma, 17-500). Samples were incubated with either anti-tryptase-6 or anti-rabbit IgG antibody (Cell Signaling Technology, Danvers, MA, USA, 7074S) as a negative control. To confirm enzymatic activity of the IP tryptase-6 eluate, 10 µl of eluate was incubated with 1 mM of VPR-AMC and assay buffer (PBS, pH=7.5) in 96-well plates in duplicate. Fluorescence release was measured as done above for the pull-down assay. All samples were run in duplicate and background fluorescence (buffer only) was subtracted. The resulting fluorescent activity was normalized to total protein levels in the eluate. For calcium signaling experiments, the FU/min/ng of tryptase-6 in the volume used is indicated. To confirm the presence of tryptase-6, the eluate and SF from the same patients were visualized by Western blot analysis as described above.

### Calcium Cell Signaling Assay

Cultured PAR-responsive human embryonic kidney (HEK)-293 cells were used to establish the ability of SF samples to activate PAR2, as described elsewhere (52). HEK-293 cells were cultured to confluence in DMEM medium containing high glucose and L-glutamine (Millipore Sigma, D5796) supplemented with 10% fetal bovine serum (FBS, Thermo Fisher Scientific, 12484028) and 100 U/ml penicillin/streptomycin (Thermo Fisher Scientific, 15140122). Cells were plated in black 96-well plates at 40,000 cells per well and left to stabilize overnight in a 37°C/5% CO_2_ incubator. The calcium-regulated fluorescent intracellular calcium indicator, Fluo-4 AM (Thermo Fisher Scientific, F36206) was used to monitor real-time elevations of intracellular calcium following activation or inhibition of PAR2, according to the manufacturer’s instructions. The PAR2 activating peptide, 2-furoyl-LIGRLO-NH2 (2fLI) and the selective PAR-2 inhibitor, I-191 [International Publication No. WO 2015/048245A1 (PCT/US2014/057390)], were used (53–55). Fluorescence was normalized to the signal generated by the calcium ionophore A23187 used at a concentration of 2 µM (Millipore Sigma, C7522). Calcium ionophore was used as a positive control to reflect calcium signaling by Fluo-4-loaded cells, as done previously (56). Data were acquired on a FLUOstar Omega plate reader at 37°C with excitation at 494 nm and emission at 516 nm (BMG Labtech, Ortenberg, Germany).

Calcium signaling in CRISPR-eliminated HEK-293 PAR2 knockout cells, generated in keeping with a previously published procedure (57), was monitored as described elsewhere (58). Fluorescence was normalized to the response caused by 2 µM calcium ionophore. Data were generated using a Perkin-Elmer spectrophotometer with excitation at 480 nm and emission at 530 nm with recordings every 0.5 sec for 1 min.

Human macrophage-related THP-1 cells (ATCC, Manassas, VA, USA) were used to test PAR2 signaling in a monocyte/macrophage cell model. Cells were seeded at 50,000 cells per well in black 96-wells and cultured in RPMI media (Thermo Fisher Scientific, 11875093) supplemented with 10% FBS, 1 mM sodium pyruvate (Thermo Fisher Scientific, 11360070), 100 U/ml penicillin/streptomycin and 25 nM of phorbol 12-myristate 13-acetate (PMA, Millipore Sigma, P8139) for 72 h to induce differentiation of cells into macrophages. Calcium signaling after activation with 2fLI and PsA SF-derived tryptase-6 and/or inhibition with I-191 was measured as described in HEK-293 cells.

Primary monocytes/macrophages were obtained from fresh SF and blood samples from PsA patients. Due to the difficulty of obtaining sufficient number of fresh SF samples, blood samples from PsA patients not receiving treatment with biologic therapy were used to complement the experiments from fresh SF samples. Peripheral blood mononuclear cells (PBMCs) were isolated from heparinized blood by Ficoll-Hypaque gradient density separation. CD14+ monocytes/macrophages were obtained from SF cells and PBMCs by magnetic-activated cell sorting (MACS) using positive selection for CD14, according to the manufacturer’s instructions (Miltenyi Biotec, Bergisch Gladbach, Germany, 130-050-201). Blood cells were further processed to select for the PAR2-expressing monocytes/macrophages by staining cells with PAR2-PE antibody (Santa Cruz Biotechnology) coupled with indirect labelling and separation of cells by positive selection using anti-PE microbeads (Miltenyi Biotec, 130-105-639). Cells were plated at 100,000 cells/well in poly-D-lysine coated black 96-well plates in RPMI medium containing L-glutamine supplemented with 10% human AB serum (Thermo Fisher Scientific, ICN2930949) and 100 U/ml penicillin/streptomycin for 48 h before performing calcium signaling experiments.

### Multiplex Cytokine Assay and ELISA

Fresh blood-acquired PAR2-expressing monocytes/macrophages were isolated from PsA patients (n=8) as described above and seeded at 100,000 cells per well in 24-well plates in RPMI media containing 10% human AB serum and 100 U/ml penicillin/streptomycin. Cells were cultured for 48 h in the presence or absence of 100 µM of 2fLI. Due to the low levels of cytokines released from unstimulated cells (59), 2.5 ng/ml of LPS (Millipore Sigma, L2630) was added during the last 4 h to induce cytokine expression. LPS is a TLR4 agonist that is recognized as a potent activator of monocytes/macrophages (60). Conditioned medium and harvested cells were stored at -80°C until analysis. Samples were screened for the expression of 20 chemokines/cytokines (MCP-1, MIP-1α, MIP-1β, CXCL1, CXCL2, CXCL10, G-CSF, GM-CSF, IFNα, IFNγ, IL-1β, IL-4, IL-6, IL-8, IL-10, IL-12p70, IL-17/IL-17A, IL-17E, PD-L1, TNFα) using the Luminex Performance Human XL Cytokine Discovery Magnetic Panel, according to the manufacturer’s instructions (R&D Systems, Minneapolis, MN, USA, FCSTM18-20).

Additional blood-derived PAR2-expressing monocytes/macrophages were isolated from PsA patients (n=6) for measurement of monocyte chemoattractant protein-1 (MCP-1) in the presence or absence of 2fLI and I-191. Cells were isolated and stored at −80°C until ready for analysis. Cells were thawed rapidly at 37°C and cultured untreated or in the presence of 100 µM 2fLI, 30 µM I-191 or both for 48 h and stimulated with LPS during the last 4 h. Total MCP-1 levels were measured in conditioned medium using the Human MCP-1 Quantikine ELISA, according to the manufacturer’s instructions (R&D Systems, DCP00).

### Statistical Analysis

The Kolmogorov–Smirnov normality test was performed for all analyses, and values were predominantly found not to be normally distributed. When comparing multiple groups, the Kruskal-Wallis test with Dunn’s multiple comparisons test or Mann Whitney U test were used. When comparing paired values, the Wilcoxon matched-pairs signed rank test was used. Statistical analysis was performed using GraphPad Prism (version 8.0.1). For all the statistical tests, p<0.05 was accepted as significant.

## Results

### Characterization of Monocyte/Macrophage Cell Subtypes in Synovial Fluid

**Figure 1:**
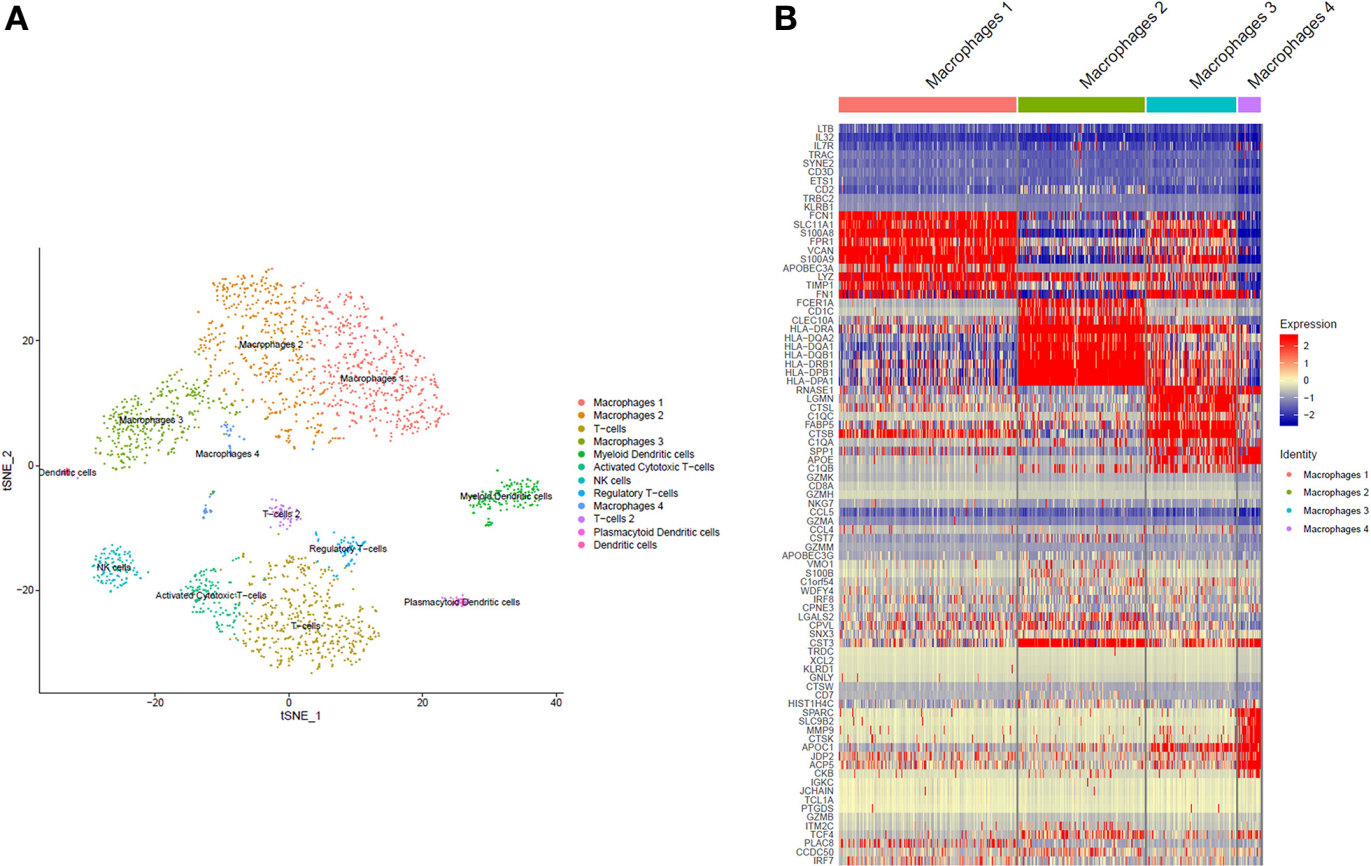
Single cell RNA sequencing. The clustering of predicted cell subpopulations from SF cells of 3 PsA patients identified 12 different cell populations, with the most dominant being monocytes/macrophages **(A)**. The monocytes/macrophages were comprised of four subpopulations, three of which were large (macrophages 1, 2 and 3) with expression levels of unique cell markers and transcriptomic signatures. The expression of top differentially expressed genes within the monocyte/macrophage subpopulations is shown in **(B)**.

To identify the cell populations in PsA SF, single cell 3’-RNA-sequencing of cells from PsA SF samples (n=3) was performed using the 10X Genomics platform (42, 43). Twelve main clusters of cells were predicted (**Figure 1A**) and the most abundant clusters had a predominance of monocytes/macrophages gene expression (differential heatmap shown in **Figure 1B**). Neutrophil transcriptomic signatures could not be detected due to low cell viability.

**Figure 2:**
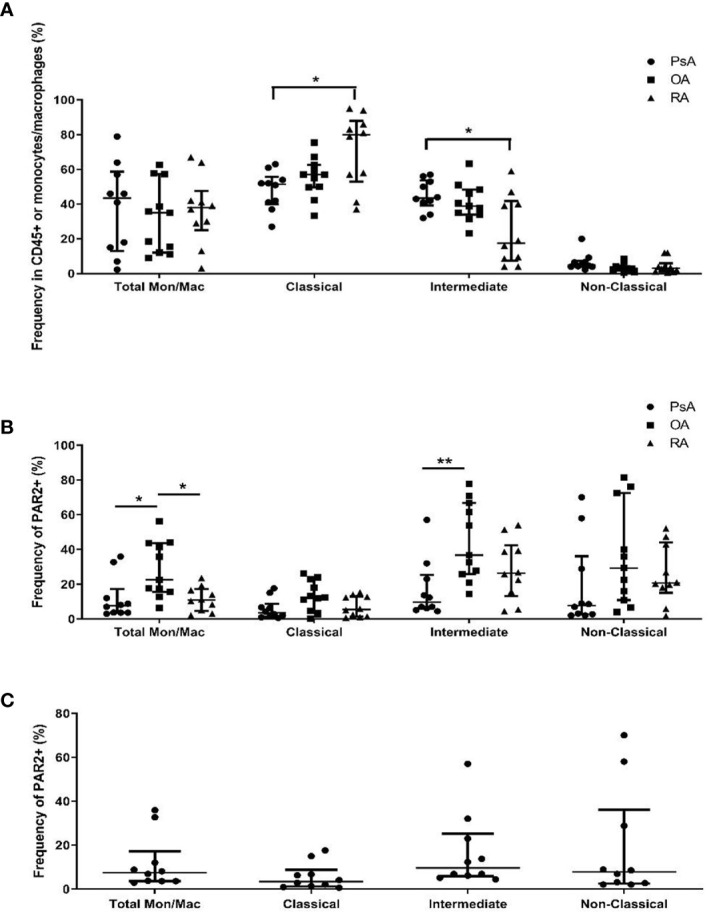
Identification of specific monocyte/macrophage subtypes and their expression of PAR2 in SF of PsA, OA and RA patients. Flow cytometry was used to determine the total **(A)** and PAR2-expressing **(B)** monocytes/macrophages from SF of PsA, OA and RA patients (n=10 each). Classical (CD14+CD16-) monocytes/macrophages are elevated in RA compared to PsA (p=0.030) while intermediate (CD14+CD16+) cells are higher in PsA compared to RA (p=0.038). PAR2 expression is increased in OA compared to PsA (p=0.014) and RA (p=0.046) patients and is expressed predominantly in cells with an intermediate phenotype (p=0.008). No significant differences are observed in PAR2 expression between cell subpopulations of PsA SF cells **(C)**. Populations were compared using the Kruskal-Wallis test with Dunn’s multiple comparisons test. Asterisks are used to indicate significant differences between groups, where *p<0.05 and **p<0.01.

The total monocyte/macrophage populations in SF as well as their expression of PAR2 were compared between PsA, RA and OA patients by flow cytometry (n=10 each). Overall monocyte/macrophage populations were similar between patient groups, but differences were identified in subset populations within PsA and RA. CD14+C16- classical monocytes/macrophages were elevated in RA compared to PsA SF (p=0.030), while CD14+CD16+ intermediate monocytes/macrophages were more predominant in PsA compared to RA SF (p=0.038, **Figure 2A**). PsA or RA monocyte/macrophage subset populations were not significantly different compared to OA SF. When we compared the expression of PAR2 within the cell populations, OA patients had elevated levels of PAR2 in the total monocyte/macrophage group compared to both PsA (p=0.014) and RA (p=0.046), evident particularly in the intermediate monocyte/macrophage subset (p=0.008 vs. PsA; **Figure 2B**). No significant differences in PAR2 expression between monocyte/macrophage subsets were observed in PsA patients (**Figure 2C**).

### Identification of Serine Proteinases in Synovial Fluid

**Figure 3:**
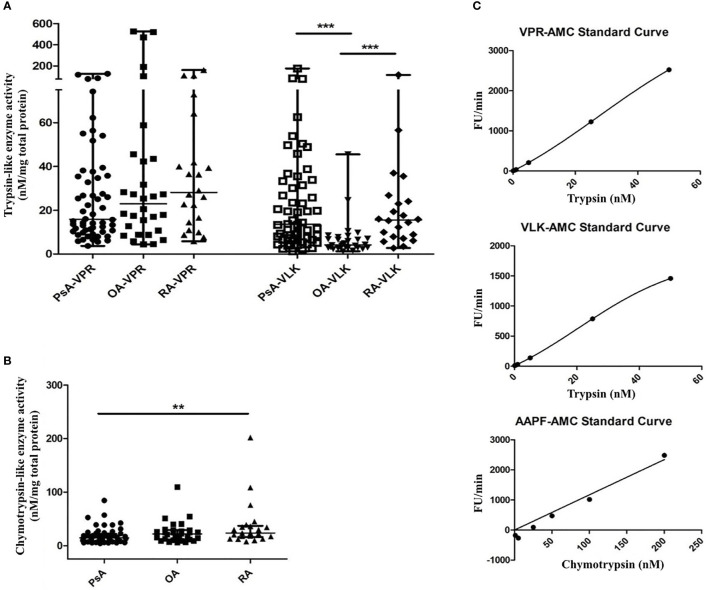
Serine proteinase activity in SF from PsA, RA and OA patients. Serine proteinase activity using fluorogenic substrates for trypsin-like proteinases with a preference for arginine (VPR) or lysine (VLK) **(A)** and chymotrypsin-like proteinases **(B)** is shown. Fluorescence release was monitored for 20 min of a kinetic cycle and the slope of the resulting curve was extrapolated relative to a standard curve of fluorescence release by known concentrations of either trypsin or chymotrypsin and normalized to the total protein concentration of the sample. Representative standard curves are shown in **(C)** for each substrate. Groups were compared using the Kruskal-Wallis test with Dunn’s multiple comparisons test. Asterisks are used to indicate significant differences between groups, where **p<0.01 and ***p<0.001.

Investigations of serine proteinase activity in samples from PsA patients have not yet been performed. This unmet need, together with our finding that serine proteinases which signal through PAR2 can be present in PsA SF (22) and combined with the availability of various tools for detecting total serine proteinase activity, led us to focus on this group of proteinases for further study. The presence of serine proteinase activity in SF samples from patients with PsA, RA and OA was confirmed by a fluorogenic proteinase substrate activity assay. A significantly higher level in trypsin-like activity of serine proteinases with a preference for lysine (VLK-AMC substrate) was found in SF from patients with PsA (median 13.51 nM/mg total protein, range 6.58–32.84, p <0.0001) and RA (median 15.57 nM/mg total protein, range 7.73–24.73, p=0.0002) compared to OA (median 4.21 nM/mg total protein, range 2.89–7.28). No significant differences in trypsin-like activity of serine proteinases with a preference for arginine (VPR-AMC substrate) were found between patient groups (**Figure 3A**). Chymotrypsin-like activity was higher in RA (median 23.70 nM/mg total protein, range 16.29–37.03) compared to PsA SF (median 14.55 nM/mg total protein, range 8.59–22.10, p=0.005), with OA SF levels (median 21.93 nM/mg total protein, range 11.40–29.21) not significantly different to either RA (p=0.718) or PsA SF levels (p=0.129; **Figure 3B**). Representative standard curves of active trypsin and chymotrypsin for each substrate are shown in **Figure 3C**.

Next, serine proteinases that might prove to be active in the SF were identified by in-gel trypsin digestion of all gel bands identified by Coomassie blue staining to capture as much of all proteinase forms present, followed by identification using mass spectrometry analysis. After filtering out data for known common contaminants (e.g. keratin), a total of 756 proteins were identified, 42 of which were potentially biologically active. There were proteinases identified which were unique to each patient group, and 25 proteinases overlapped between all three groups. The full list of identified proteinases containing proteinase domains and listed as biologically active in the *homo sapiens* MEROPS database (61) is shown in **Table 1**. There were 3 proteinase-related proteins that were unique to PsA patients, including the cysteine proteinase bleomycin hydrolase [BLMH], the pseudoprotease sequence-containing sonic hedgehog protein [SHH] and the aspartic acid proteinase cathepsin D (CTSD). Overall, the majority of the identified entries were serine proteinases which were primarily involved in the coagulation and complement cascade. The remaining serine proteinases identified were evaluated according to their potential biological function, particularly in relation to monocytes/macrophages given their prevalence in PsA SF. Tryptase-6 (PRSS33), originally termed EOS, was of particular interest due to its predicted molecular weight falling in the range of known tryptic enzymes, its presence in all patient samples and a previous report which first identified tryptase-6 as a novel serine proteinase expressed predominantly by macrophages (62).

**Table 1:** List of proteins with proteinase sequences identified in SF of PsA, OA and RA patients.

Gene Name	Full Name	Type	UniProt ID	MEROPS ID^1^	Human Disease
CTSD	Cathepsin D	Aspartic	P07339	A01.009	PsA
CASP14	Caspase-14	Cysteine	P31944	C14.018	RA, OA
CTSZ	Cathepsin X	Cysteine	Q9UBR2	C01.013	PsA, RA
GGH	Gamma-glutamyl hydrolase	Cysteine	Q92820	C26.001	PsA, OA, RA
CTSS	Cathepsin S	Cysteine	P25774	C01.034	PsA, OA, RA
CTSB	Cathepsin B	Cysteine	P07858	C01.060	PsA, OA, RA
SHH	Sonic hedgehog protein	Cysteine	Q62226	C46.002	PsA
BLMH	Bleomycin hydrolase (animal)	Cysteine	Q13867	C01.084	PsA
CPB2	Carboxypeptidase B2	Metallo	Q96IY4	M14.009	PsA, OA, RA
LAP3	Cytosol aminopeptidase	Metallo	P28838	M17.001	PsA, RA
MMP8	Neutrophil collagenase	Metallo	P22894	M10.002	RA
MMP3	Matrix metallopeptidase-3	Metallo	P08254	M10.005	PsA, RA
MMP1	Interstitial collagenase	Metallo	P03956	M10.001	RA
MMP2	Matrix metallopeptidase-2	Metallo	P08253	M10.003	PsA, OA, RA
MMP9	Matrix metalloproteinase-9	Metallo	P14780	M10.004	RA
NPEPPS	Puromycin-sensitive aminopeptidase	Metallo	P55786	M01.010	RA
ANPEP	Aminopeptidase N	Metallo	P15144	M01.001	PsA, OA, RA
ACE	Angiotensin-converting enzyme	Metallo	P12821	M02.001	RA
CFD	Complement factor D	Serine	P00746	S01.191	PsA, OA, RA
PRTN3	Myeloblastin	Serine	P24158	S01.134	PsA, RA
ELANE	Elastase-2	Serine	P08246	S01.131	RA
CTSG	Cathepsin G	Serine	P08311	S01.133	RA
PRSS33	Tryptase-6	Serine	Q8NF86	S01.075	PsA, OA, RA
CFH	Complement factor H	Serine	P08603	M43.UNB	PsA, OA, RA
F9	Coagulation factor Ixa	Serine	P00740	S01.214	PsA, OA, RA
PROC	Vitamin K-dependent protein C/Protein C (activated)	Serine	P04070	S01.218	PsA, OA, RA
CTSA	Lysosomal protective protein	Serine	P10619	S10.002	RA
HABP2	Factor VII-activating peptidase	Serine	Q14520	S01.033	PsA, OA, RA
CFI	Complement factor I	Serine	P05156	S01.199	PsA, OA, RA
F12	Coagulation factor XIIa	Serine	P00748	S01.211	PsA, OA, RA
F2	Thrombin	Serine	P00734	S01.217	PsA, OA, RA
F11	Coagulation factor Xia	Serine	P03951	S01.213	PsA, OA, RA
HGFAC	Hepatocyte growth factor activator	Serine	Q04756	S01.228	PsA, OA, RA
KLKB1	Plasma kallikrein	Serine	P03952	S01.212	PsA, OA, RA
C1S	Complement component activated C1s	Serine	P09871	S01.193	PsA, OA, RA
LTF	Lactoferrin	Serine	P02788	S60.001	PsA, OA, RA
MASP1	Mannan-binding lectin-associated serine peptidase-3	Serine	P98064	S01.132	PsA, OA, RA
C1R	Complement component activated C1r	Serine	P00736	S01.192	PsA, OA, RA
C2	Complement component C2a	Serine	P06681	S01.194	PsA, OA, RA
CFB	Complement factor Bb	Serine	P00751	S01.196	PsA, OA, RA
DPP4	Dipeptidyl-peptidase IV	Serine	P27487	S09.003	PsA, RA
PLG	Plasmin	Serine	P00747	S01.233	PsA, OA, RA

^1^Proteinases reported as biologically active in Homo sapiens MEROPS database that contain proteinase domains are listed.

### Tryptase-6 Is an Active Serine Proteinase in Synovial Fluid

**Figure 4:**
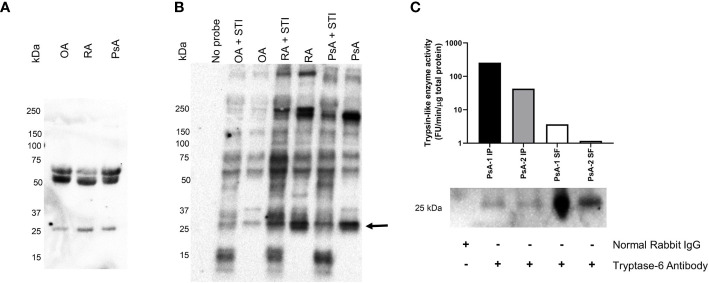
Synovial fluid tryptase-6 aligns with active serine proteinases from PsA, OA and RA patients. Tryptase-6 was detected by western blot analysis of individual SF samples from PsA, OA and RA patients **(A)**. In parallel, trypsin-like serine proteinase activity was identified in SF samples from the same individuals after covalent labelling with a biotinylated ABP probe for trypsin-like serine proteinases in the presence or absence of the trypsin inhibitor, STI (4 µg/ml), and visualization by western blot detection using streptavidin-HRP **(B)**. An ABP-labelled band at the predicted molecular weight of active tryptase-6 (~25 KDa), for which labelling was reduced in the presence of STI was observed (arrow, **B**). A column-based antibody affinity chromatography procedure was used to isolate tryptase-6 from PsA SF samples (n=2). The activity of the isolated enzyme and its identity were confirmed by its ability to cleave the substrate, VPR-AMC, and by western blot detection with a tryptase-6 antibody, respectively **(C)**.

Our next step was to determine if tryptase-6 was present in SF as an active enzyme. To this end, we used western blot analysis using a tryptase-6-targeted antibody and an antibody affinity column to capture and isolate active enzyme from the SF samples. A serine proteinase-targeted biotinylated activity-based probe that selectively reacts with the active serine in trypsin-like proteinases was also used to identify active serine proteinases in the SF samples. Upon covalent reaction with the proteinase, this probe enables the visualization of the active biotinylated enzyme by an avidin-based western blot procedure. The activity-based probe labelling was done in the absence and presence of soybean trypsin inhibitor (STI) to determine if the proteinases in SF were active and not binding to the activity-based probe non-specifically. The western blot analysis was done with a polyclonal antibody specific for tryptase-6 which reacts with an epitope in the N-terminal region of tryptase-6 (within amino acids 30–70), allowing potentially for the detection of both active and non-active forms of the enzyme (62). Western blots analysis was done using OA, RA and PsA SF samples (n=3), with representative blots for the enzyme detected by the tryptase-6 antibody shown in **Figure 4A**. Based on molecular weight, antibody reactivity was observed for all three patient-derived samples in the 25 kDa region, as expected for the active form of tryptase-6 (**Figure 4A**). Antibody reactivity was also seen for all three samples in the higher molecular mass region of the gel (50 to 70 kDa: **Figure 4A**). These proteins may represent an enzyme dimer or a larger inactive enzyme form bound to a proteinase inhibitor. A biotin signal localizing a proteinase tagged by the biotinylated serine proteinase-selective activity-based probe was also observed in the 25 kDa region of the gel similar to an enzyme with the mobility of tryptase-6 (**Figure 4B**). The signal in the 25 kDa region of the gel was clearly reduced by STI in samples from the RA and PsA patients; less so in the sample from the OA individual. An activity-based-probe-labelled enzyme with a reduced signal in the presence of STI was also observed in the 200 kDa range for the RA and PsA samples; and an STI-inhibited enzyme with a mass > 250 kDa was also observed in the RA sample (**Figure 4B**). The biotinylation of many other activity-based-probe-labelled enzymes was not affected by STI (**Figure 4B**), pointing to a wide spectrum of active enzymes not susceptible to STI inhibition. The tryptase-6 antibody also visualised a 25 kDa protein eluted from the antibody affinity beads that captured the protein from the PsA-derived SF samples (**Figure 4C**). This result indicated that the tryptase antibody affinity column could interact with enzymatically active enzyme which could be eluted for further analysis (compare histograms in the upper panel of **Figure 4C** with the blots in the lower panel).

**Figure 5:**
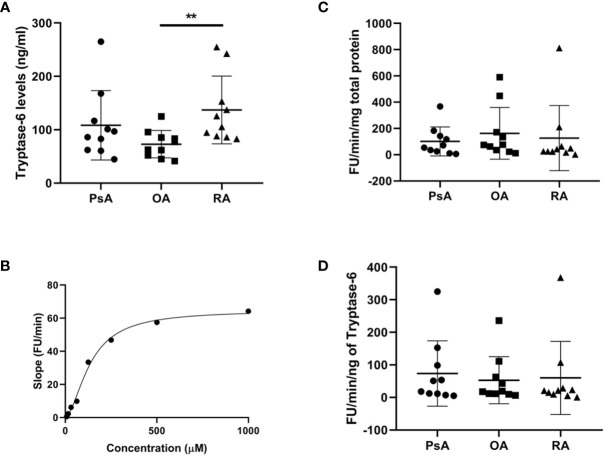
Total and active tryptase-6 levels detected in SF from PsA, OA and RA patients. Total and active tryptase-6 levels were determined by a commercially available ELISA and a pull-down activity assay, respectively in PsA, OA and RA SF samples (n=10 per group). As shown in **(A)**, total levels of tryptase-6 were elevated in RA (median 115.6 ng/ml, range 87.6-175.5) compared to OA SF (median 70.08 ng/ml, range 50.3–87.9, p=0.002, Mann Whitney U test). The activity of pulled down-tryptase-6 from PsA SF towards VPR-AMC generated the substrate cleavage curve shown in **(B)**. Tryptase-6 activity from SF samples, after normalization to either total protein levels **(C)** or total tryptase-6 **(D)**, did not reveal any significant differences between groups. Asterisks are used to indicate significant differences between groups, where **p<0.01.

After detecting active tryptase-6 in the affinity column-isolated SF samples, we used a more targeted and quantitative approach to compare total immunoreactive and enzymatically active levels of tryptase-6 in SF from PsA, RA and OA patients (n=10 each). As shown in **Figure 5A**, the total levels of tryptase-6 measured by the ELISA were higher in RA (median 115 ng/ml, range 87.6–175.5) compared to OA SF (median 70.1 ng/ml, range 50.3–87.9, p=0.002), but not PsA-derived samples (median 91.6 ng/ml, range 61.8–129.4, p=0.248). Using a pull-down assay, active tryptase-6 in SF was quantified by its incubation with varying concentrations of the fluorogenic substrates for serine proteinases with a preference for arginine (VPR-AMC) and lysine (VLK-AMC). Tryptase-6 activity increased when both substrates were added, with the resulting substrate cleavage curve shown for VPR-AMC in **Figure 5B**. The specific activity levels of tryptase-6 measured with the VPR-AMC substrate for SF samples from the PsA, RA, and OA patients are shown normalized to total protein levels (**Figure 5C**) and tryptase-6 levels (**Figure 5D**). No significant differences were observed between the three patient groups. Taken together, these results confirm that active tryptase-6 is present in arthritic SF samples at comparable levels of enzyme activity.

### Tryptase-6 Triggers Calcium Signals in HEK-293 Cells Partially *via* PAR2

**Figure 6:**
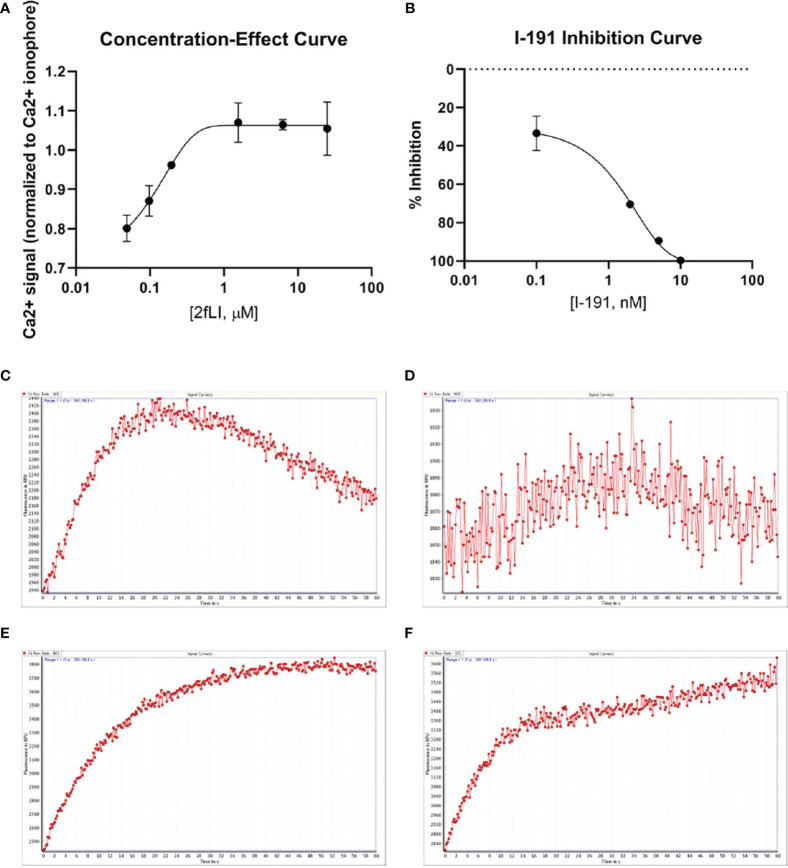
PAR2 and tryptase-6 trigger calcium signals in HEK-293 cells. Calcium signaling assay using the fluorescent indicator, Fluo-4 AM, was used to confirm the signaling potential of PAR2 and determine whether tryptase-6 can elicit a calcium response *via* PAR2 in HEK-293 cells. The concentration-effect curve for the PAR2 agonist 2fLI with an EC50 of 0.14 µM **(A)** and the concentration–inhibition curve for the PAR2 inhibitor I-191 with an IC50 of 2.1 nM **(B)** are shown. Stimulation of cells with 0.15 µM of 2fLI **(C)** was inhibited 99% by 10 nM of I-191 **(D)**. Stimulation of cells with 10.0 FU/min/ng tryptase-6 isolated by antibody affinity chromatography from PsA SF caused an elevation of intracellular calcium **(E)** and 10 nM of I-191 caused a 34% inhibition of this signal **(F)**. Calcium traces are shown with agonists (2fLI and tryptase-6) added at time zero with or without pre-treatment with I-191 (x-axis=time post addition of agonist, y-axis=units of fluorescence due to calcium release).

**Figure 7:**
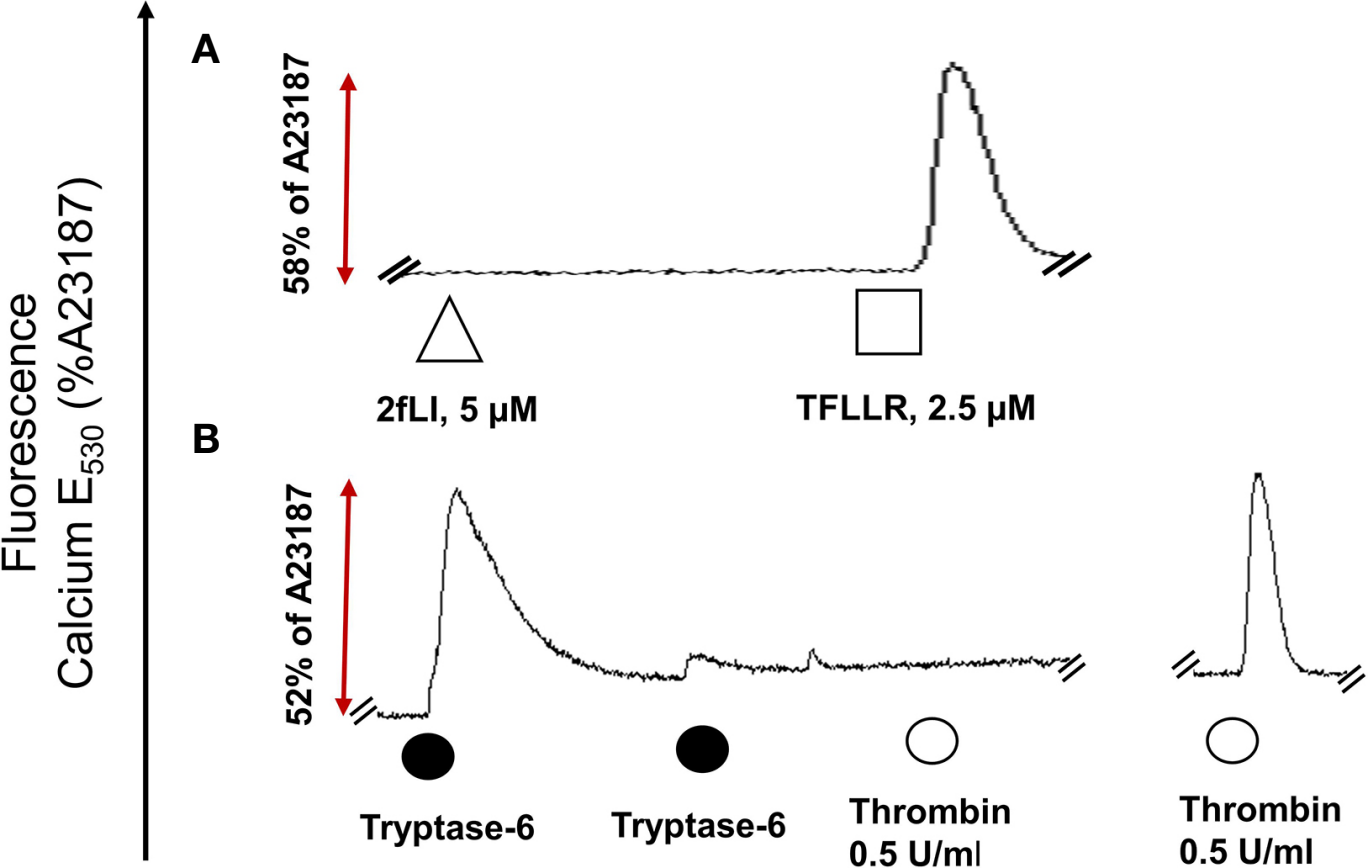
Tryptase-6 triggers calcium signals in PAR2 knockout HEK-293 cells. The calcium signaling assay [fluorescence emission at 530 nm, as a percentage of the signal generated by the calcium ionophore A23187 (%)] was used to determine whether tryptase-6 can trigger calcium signaling *via* PAR2-independent pathways. Tracing **(A)**: The absence of a response to the potent PAR2-selective agonist, 2fLI (open triangle) but presence of a response to the selective PAR1 peptide agonist, TFLLR-amide (open square) confirmed the absence of functional PAR2 and the presence of functional PAR1 in the HEK-293-PAR2-null cells. Tracing **(B)**: Volume equivalent to 5.8 FU/min/ng of PsA SF-derived tryptase-6 (solid circle) elicited a robust calcium response (dark circle), that was desensitized by the second consecutive exposure to tryptase-6 (second solid circle, Tracing **B**). These tryptase-6-treated cells no longer responded to the PAR1 activator, thrombin (0.5 U/ml, open circle, first tracing panel **B**). Cells not treated with tryptase-6 showed a strong calcium signal caused by thrombin (right-hand tracing, panel **B**).

The next goal was to investigate whether active tryptase-6 could stimulate a calcium signaling response in intact cells and to determine whether this response was mediated by PAR2. Initially the widely-used PAR-responsive cell line, HEK-293, was used. Treatment of HEK-293 cells with the PAR2 peptide agonist (2fLI) caused a concentration-dependent increase in calcium signaling with an EC50 of 0.14 µM (**Figure 6A**). As shown in **Figure 6B**, the PAR2-selective inhibitor, I-191, successfully blocked the PAR2 calcium response when cells were pre-treated with increasing concentrations of I-191, followed by 0.15 µM of 2fLI. The IC50 for the ability of I-191 to block PAR2 signaling was 2.1 nM (**Figure 6B**). Thus, stimulation of wild-type HEK cells with 0.15 µM of 2fLI (**Figure 6C**) was inhibited 99% by 10 nM of I-191 (**Figure 6D**). Treatment of the same wild-type HEK cells with 10.0 FU/min/ng tryptase-6 isolated from the PsA SF by antibody affinity chromatography caused an increase in intracellular calcium (**Figure 6E**) which was inhibited 34% by 10 nM of I-191 (**Figure 6F**). In addition, calcium signaling upon stimulation with tryptase-6 was assessed in CRISPR-eliminated PAR2 knockout HEK-293 cells (**Figure 7**). The PAR2-null cells were not responsive to the potent selective PAR2 peptide agonist, 2fLI (5 µM), but were still sensitive to 2.5 µM of the receptor-selective PAR1 agonist, TFLLR-amide (**Figure 7**, tracing A). In these PAR2-null/PAR1-expressing HEK cells, treatment with 5.8 FU/min/ng of PsA SF-derived tryptase-6 caused an elevation of intracellular calcium that was desensitized to the second challenge with tryptase-6. The tryptase-6-desensitized cells no longer responded to a PAR1-associated response caused by stimulation with 0.5 U/ml of thrombin (open circle, first tracing in **Figure 7B**). In contrast, a robust response to thrombin was observed for the cells not pre-treated with tryptase-6 (right-hand tracing, open circle, **Figure 7B**). Thus, tryptase-6 was able to prevent thrombin’s ability to activate PAR1 either by signal desensitization or by removing the PAR1 tethered ligand so as to “disarm” PAR1. The ability of tryptase-6 to activate MAPKinase signaling was also assessed in HEK-293 cells by western blot analysis. In keeping with the calcium signaling data, western blot detection of activated phospho-MAPKinase caused by tryptase-6 was able to demonstrate the activation of MAPKinase in both PAR2-expressing and PAR2-null HEK cells (data not shown). These results indicate that tryptase-6 stimulates both calcium and MAPKinase signaling in HEK-293 cells not only *via* PAR2 but also *via* a PAR2-independent mechanism. Therefore, in the microenvironment of the SF containing tryptase-6, multiple receptors on monocytes/macrophages or other cell types in principle can be activated by this proteinase, including PAR2.

### Tryptase-6 Triggers Calcium Signals in THP-1 Cells and Primary Monocytes/Macrophages Partially *via* PAR2

**Figure 8:**
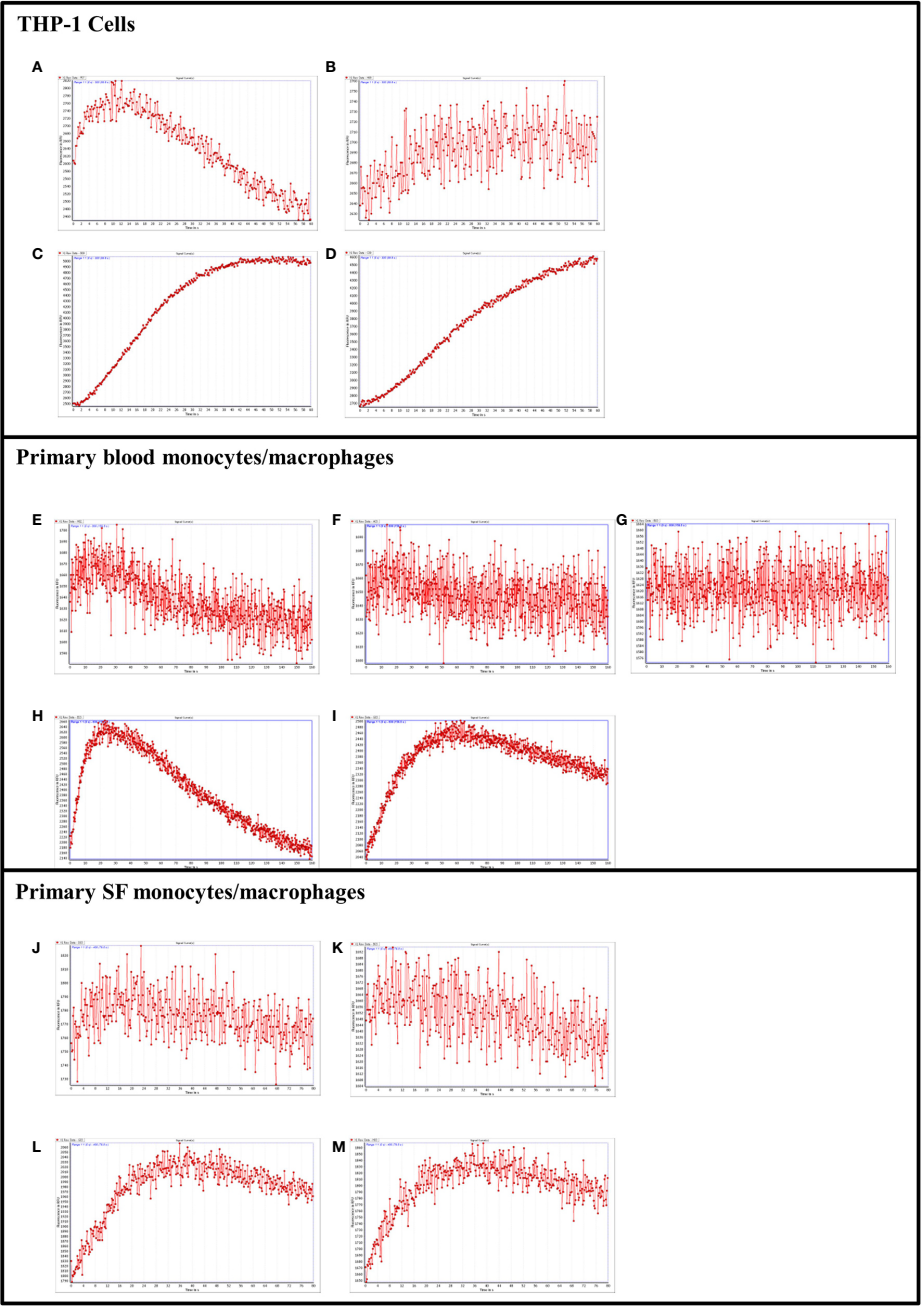
PAR2 and tryptase-6 trigger calcium signals in THP-1 cells and PsA PAR2+ monocytes/macrophages. Calcium signaling assay using the fluorescent indicator, Fluo-4 AM, was used to confirm the signaling potential of PAR2 and determine whether tryptase-6 can elicit a calcium response *via* PAR2 in THP-1 cells and primary monocytes/macrophages from PsA patients. Stimulation with 2fLI in THP-1 (150 µM, **A**) or primary blood PAR2+ monocytes/macrophages (100 µM, **E**) was inhibited 12% **(B)** and 8% **(F)** by 1 µM of I-191, respectively. In blood cells, 2 µM of I-191 inhibited this signal by 27% **(G)**. Stimulation of THP-1 cells **(C)** and primary cells **(H)** with volume equivalent to 44.8 or 89.6 FU/min/ng tryptase-6 isolated with the antibody affinity column from PsA SF, respectively, caused an elevation of intracellular calcium. Treatment with 1 µM of I-191 caused a 15% **(D)** and 13% **(I)** inhibition of this signal, respectively. Similarly, in primary SF monocytes/macrophages, 2fLI (200 µM, **J**) and volume equivalent to 44.8 FU/min/ng tryptase-6 **(L)** caused a calcium flux that was inhibited 51% **(K)** and 40% **(M)** by 1 µM of I-191, respectively. Calcium traces are shown with agonists (2fLI and tryptase-6) added at time zero with or without pre-treatment with I-191 (x-axis=time post addition of agonist, y-axis=units of fluorescence due to calcium release).

In keeping with the data obtained for the HEK-293, THP-1 cells were used to establish the ability of tryptase-6 to activate calcium signaling in monocytes/macrophages *via* PAR2. THP-1 cells were partially differentiated into macrophages using PMA to increase endogenous PAR2 expression (63, 64). Concentrations of 50 to 200 µM of 2fLI produced small but reliable increases in intracellular calcium in the cells. In **Figure 8A**, 150 µM of 2fLI induced calcium release that was inhibited 12% by 1 µM of the potent PAR2-selective inhibitor I-191 (**Figure 8B**). Calcium flux by stimulation with 44.8 FU/min/ng tryptase-6 eluate (**Figure 8C**) was also inhibited 15% by I-191 (**Figure 8D**). Similarly, treatment of peripheral blood-derived PAR2-expressing monocytes/macrophages from PsA patients with 100 µM of 2fLI caused an increase in intracellular calcium (**Figure 8E**), which was inhibited 8% by 1 µM (**Figure 8F**) and 27% by 2 µM of I-191 (**Figure 8G**). Treatment of cells with 89.6 FU/min/ng tryptase-6 isolated from PsA SF also caused an elevation of intracellular calcium (**Figure 8H**) that was inhibited 13% by 1 µM of I-191 (**Figure 8I**). In PsA SF-isolated monocytes/macrophages, 200 µM of 2fLI caused an increase in intracellular calcium (**Figure 8J**), which was inhibited 51% by 1 µM of I-191 (**Figure 8K**). Treatment of cells with 44.8 FU/min/ng tryptase-6 derived from PsA SF also caused an elevation of intracellular calcium (**Figure 8L**) that was inhibited 40% by 1 µM of I-191 (**Figure 8M**). These data indicate that tryptase-6 mediates calcium signaling in PsA monocytes/macrophages in part *via* PAR2. Furthermore, PsA SF monocytes/macrophages seem more responsive to PAR2 activation, including calcium signalling responses triggered by tryptase-6, compared to peripheral monocytes/macrophages.

### PAR2 Modulates Monocyte/Macrophage MCP-1 Production

**Figure 9:**
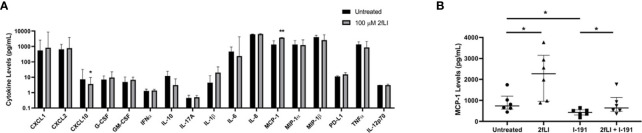
PAR2 modulates MCP-1 expression from PsA monocytes/macrophages. The expression of 20 chemokines/cytokines measured from conditioned medium of blood-derived PAR2-expressing monocytes/macrophages of PsA patients (n=8) using a multiplex Luminex assay is shown in **(A)**. Cells were cultured for 48 h in the presence or absence of 100 µM 2fLI and stimulated with LPS during the last 4 h. The expression of IFNγ, IL-17E and IL-4 were below the limits detection and are not shown. Values are graphed on a log_10_ scale to better visualize differences between groups. MCP-1 was also measured in additional samples of blood-derived PAR2-expressing monocytes/macrophages (n=6) in the presence of 30 µM I-191, 100 µM 2fLI or both **(B)**. Expression was compared between groups using the Wilcoxon matched-pairs signed rank test. Error bars indicate the median ± interquartile range. Asterisks are used to indicate significant differences between groups, where *p < 0.05 and **p < 0.01.

Blood-derived PAR2-expressing monocytes/macrophages from PsA patients (n=8) were incubated for 48 h in the presence or absence of 100 µM of the receptor-selective PAR2 agonist, 2fLI, to determine the impact of PAR2 activation on cytokine expression. The expression/secretion of chemokines/cytokines in conditioned medium is shown in **Figure 9A**. 2fLI treatment caused an increase in MCP-1 levels (median 3,748 pg/ml, range 3,504–3,815 pg/ml) compared to untreated samples (median 1,346 pg/ml, range 661–2,342 pg/ml, p=0.008). CXCL10 levels were reduced in the presence of 2fLI (median 3.6 pg/ml, range 3.1–9.5 pg/ml) compared to untreated cells (median 7.4 pg/ml, range 4.7–32.4 pg/ml, p=0.016). Of note, CXCL1 levels (median 832 pg/ml, range 389–8,706 pg/ml, p=0.078) and IL-8 levels (median 6,468, range 6,060–6,942 pg/ml, p=0.055) were also higher in 2fLI treated cells compared to untreated cells (median 549 pg/ml, range 404–2,588 pg/ml and median 6,168, range 4,319–6,526 pg/ml, respectively). The expression of IFNγ, IL-17E and IL-4 were below the limits of detection for all samples and are not shown.

Due to the significant increase in MCP-1 levels observed following 2fLI treatment, MCP-1 was measured from the conditioned medium of blood-derived PAR2-expressing monocytes/macrophages from PsA patients (n=6) in the presence or absence of 100 µM 2fLI, 30 µM I-191 or both (**Figure 9B**). MCP-1 levels were significantly elevated by the potent PAR2-selective peptide agonist, 2fLI (median 2,272 pg/ml, range 962.7–3,147 pg/ml, p=0.031) and reduced by I-191 (median 432.7 pg/ml, range 306.8–556.3 pg/ml, p=0.031) compared to untreated cells (median 738.5 pg/ml, range 586.1–1,206 pg/ml). When 2fLI was combined with I-191 (median 640.1 pg/ml, range 481.6–1,141 pg/ml), the levels were reduced to a similar level as for the untreated cells (p=1.00). In these cells, the expression of MCP-1 was marginally reduced compared to 2fLI alone (p=0.063) but still significantly elevated compared to I-191 alone (p=0.031).

### PAR2+CCR2+ Monocytes/Macrophages are Elevated in PsA Synovial Fluid Cells

**Figure 10:**
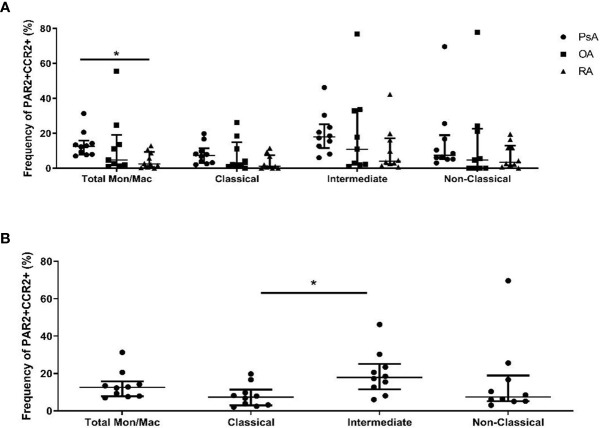
PAR2+CCR2+ monocytes/macrophages are elevated in PsA SF cells. Flow cytometry was used to determine the frequency of PAR2+CCR2+ monocytes/macrophages from SF of PsA, OA and RA patients (**A**; n=10 each). PsA patients had significantly higher total PAR2+CCR2+ monocytes/macrophages compared to RA patients (p=0.018). The proportion of total and subsets of monocytes/macrophages within PsA patients were compared in **(B)**. Intermediate (CD14 +CD16+) PAR2/CCR2 expressing cells were higher than classical (CD14+CD16-) PAR2/CCR2 expressing cells in PsA patients (p=0.001). Error bars indicate median ± interquartile range. Asterisks are used to indicate significant differences between groups, where *p<0.05.

Due to the elevated expression of MCP-1 after 2fLI treatment of PAR2+ monocytes/macrophages, we used flow cytometry to determine the expression of the receptor for MCP-1, CCR2, in monocyte/macrophage subsets and in SF cells of PsA patients and compare its expression to patients with OA and RA (n=10 each). In **Figure 10A**, the total PAR2 and CCR2-expressing monocytes/macrophages are elevated in PsA as compared to RA patients (p=0.018) but no differences in PAR2+ CCR2-expressing cells were observed in the monocyte/macrophage subsets between patient groups. Within PsA patients (**Figure 10B**), the proportion of intermediate (CD14+CD16+) PAR2+CCR2+ monocyte/macrophages were elevated compared to the classical (CD14+CD16-) PAR2+CCR2+ population (p=0.001).

## Discussion

Our data are the first to compare the differential presence of inflammatory cells (with a focus on monocyte/macrophage subtypes) and the presence of serine proteinases that could in principle regulate cell function by modulating the activity of proteinase-activated receptor 2 in PsA SF cells. We found that there were 3 main monocyte/macrophage sub-populations in PsA SF that expressed PAR2 and identified proteinases in PsA SF with the potential to regulate PAR2 signaling. Of particular note was the presence of the serine proteinase tryptase-6, known to be expressed predominantly by macrophages (62), which could in principle signal *via* both PAR2-dependent and PAR2-independent mechanisms to activate calcium signaling. We also observed increased levels of MCP-1 from PsA patient-derived monocytes/macrophages in response to PAR2 activation and increased CCR2-expressing SF monocytes/macrophages in PsA, highlighting a potential mechanism of PAR2-mediated recruitment of monocytes/macrophages to the PsA joint.

Several SF proteinases, with particular attention paid to MMPs and cysteine proteinases, have been singled out as playing an important role in arthritis (7), mainly because of their ability to degrade joint cartilage. Serine proteinases released from joint tissue and immune cells or entering the synovial space from blood have also been suggested for their potential importance in arthritis (7). More recently, attention has turned to the ability of these proteolytic enzymes to trigger inflammatory receptor signals through PARs in the joint space (7). Studies with PAR2-null mice have shown that PAR2 can play an important role in murine models of arthritis with some evidence also supporting a role for PAR2 in human OA and RA. In murine models, F2r-/- mice that lack PAR1 have reduced degradation of cartilage and expression of cytokines and MMP13 in SF (65), while swelling was reduced after PAR2 blockade in a model of joint inflammation (30). Inhibition of PAR2 can also alter the release of pro-inflammatory cytokines in cultured synovial tissue cells from RA and OA patients (33, 66). Our study is the first to examine the possible role of PAR2 and its possible activating proteinases in PsA.

As summarized, one of the main findings of our work was that the most abundant cell clusters of inflammatory cells in PsA SF were monocytes/macrophages with three main phenotypes representing classical, non-classical and intermediate cells. Although the frequency of total monocytes/macrophages did not vary between groups, RA patients had an increased frequency of classical monocytes/macrophages compared to PsA patients, while PsA patients had predominantly an intermediate monocyte/macrophage sub-population in their SF samples. This result aligns with previous reports of pro-inflammatory cytokine production and cartilage and bone destruction by RA macrophages and the increased number of pro-inflammatory macrophages in RA patients with active disease (67, 68). Notably, it has been shown that the total number of monocytes/macrophages is equivalent between patients with RA and SpA, including PsA, but CD163+ anti-inflammatory macrophages are elevated in SpA synovitis (69, 70).

We also characterized PAR2 expression in SF monocytes/macrophages and found elevated PAR2 in OA compared to PsA/RA patients, especially within the intermediate population. Although this cell subtype-specific expression has not been previously investigated, the ability of PAR2 to modulate bone pathology and pro-inflammatory changes in OA has been documented in experimental arthritis models as well as in human synovial cells (71, 72). Previous groups have detected PAR2 on peripheral blood monocyte/macrophages from healthy donors (64) and RA and OA patients (73, 74) and within the RA synovium (66), but this is the first report of PAR2 expression within monocytes/macrophages in SF of PsA patients.

We were also able to show that the monocytes/macrophage populations can respond to PAR2 activation by means of calcium and MAPKinase signaling. Activation of these signaling pathways by tryptase-6 to mimic the action of the PAR2-selective peptide agonist, 2fLI, was confirmed in HEK-293 cells, which are well-recognized for their responsiveness to PAR2 stimulation with this agonist (52, 54). In monocyte/macrophage cell populations, the calcium response to 2fLI activation was small. This result is consistent with a previous report, where the low expression of PAR2 mRNA resulted in a small response to the PAR2 activating peptide (SLIGKV-amide) in monocytes that was partially increased when cells were differentiated into macrophages by GM-CSF or M-CSF (64). Stimulation of macrophage-related THP-1 cells with phorbol ester (PMA) and isolation of PAR2+ monocytes/macrophages from PsA patients also improved their responsiveness to the selective PAR2 agonist, 2fLI, in this study.

PAR2 activation also modulated expression of cytokines in PsA-derived monocytes/macrophages, with a significant increase in MCP-1 and decrease in CXCL10. Other studies have found increased expression of MCP-1, along with other pro-inflammatory cytokines such as IL-1β, IL-6, and TNFα following PAR2 activation in models such as bone marrow-derived macrophages, the murine macrophage cell line RAW264.7, A549 lung cancer cells and human vascular endothelial cells (75–77). PAR2 activation has also been observed to cause an increase in anti-inflammatory IL-10 and suppression of IL-6, IL-12, and TNFα in murine macrophages (78). These data indicate that differences in PAR2-mediated cytokine expression can occur, with both pro- and anti-inflammatory consequences (79). When examining the expression of CCR2, the receptor for MCP-1 in SF cells, PsA patients had high overall levels of CCR2 in monocytes/macrophages, particularly in intermediate CD14+CD16+ cells. The increased production of MCP-1 upon PAR2 activation in PsA monocytes/macrophages may result in recruitment of additional CCR2-expressing intermediate monocytes/macrophages to perpetuate an immune response. This intermediate population has been shown to exert either pro- or anti-inflammatory effects, depending on the setting of their activation (80, 81). PAR2-mediated polarization towards both pro- and anti-inflammatory macrophage phenotypes has also been reported, such as the pro-inflammatory polarization of murine macrophages (75) and the repair-associated response in ischemic tissues and colitis (82, 83).

In searching for potential PAR2 activating proteinases in SF, we singled out tryptase-6, a novel serine proteinase that to date has not been identified in the setting of arthritis. This trypsin-like serine proteinase was first identified in 2003 due to its similarity to prostasin and tryptases (62, 84–87). Tryptase-6 was originally found to be primarily localized to human macrophages and up-regulated upon PMA-induced activation. The amino-acid sequence of this proteinase shares 44% identity with human β-tryptase produced from mast cells that may possibly play a role in activating PAR2 (88). Tryptase-6 has been the focus of few studies, most of which have concentrated on its role in airway inflammatory diseases (89, 90). One study found up-regulation of blood tryptase-6 mRNA from patients with early-onset atopic dermatitis (91). We showed that total but not active tryptase-6 levels are elevated in patients with inflammatory arthritis, particularly RA. Furthermore, tryptase-6 isolated from PsA SF stimulates calcium signaling in a partially PAR2-dependent manner in cell lines (HEK-293 or THP-1) and primary monocytes/macrophages from PsA patients (SF or blood). Toyama et al. have also shown that tryptase-6 can increase collagen and fibronectin mRNA in human fibroblasts in part *via* activation of PAR2 (89). Our preliminary studies using CRISPR-PAR2-null HEK-293 cells confirm that tryptase-6 causes a small PAR2-mediated calcium response; but it also causes calcium signals *via* a non PAR2 target present in HEK-293 cells. Interestingly in PsA, PAR2-expressing monocytes/macrophages from SF were more sensitive to tryptase-6-mediated PAR2 calcium signaling compared to those from the periphery. Therefore, our data show not only that this enzyme is proteolytically active in PsA SF samples and is capable of regulating the activity of PAR2 but also that it may stimulate other non-PAR2 inflammatory signals.

Taken together, our data thus point to the existence of a macrophage-triggered autocrine-paracrine loop in PsA, whereby the invading inflammatory macrophages can both produce and respond to tryptase-6 *via* PAR activation, to further the disease process. Elevation of PAR2 and CCR2-expressing CD14+CD16+ cells and an increased expression of MCP-1 amongst other cytokines following PAR2 activation can mediate recruitment of further peripheral monocytes/macrophages to the joint, perpetuating the immune response (92).

In summary, we characterized the expression of PAR2 in SF monocytes/macrophages and evaluated its signaling potential within these cells. Tryptase-6 was identified as an active serine proteinase in SF that induced a partial PAR2-dependent calcium response. Treatment of PsA monocytes/macrophages with a PAR2 agonist induced expression of cytokines with significant MCP-1 production. The expression of CCR2 on SF CD14+CD16+ monocytes/macrophages indicates that this may be an important cell population for the PAR2-mediated recruitment of monocytes/macrophages to the PsA joint. Pending future studies, these results may point to novel therapeutic targets for the management of PsA.

## Data Availability Statement

The data sets presented in this study can be found in online repositories. The names of the repositories and accession number(s) can be found below: https://www.ncbi.nlm.nih.gov/geo/, GSE161500; https://www.ebi.ac.uk/pride/archive/, PXD022442.

## Ethics Statement

The studies involving human participants were reviewed and approved by University Health Network, Research Ethics Board. The patients/participants provided their written informed consent to participate in this study.

## Author Contributions

FA, MR, AG-A, CM, KM, MM, MS, and KO performed the experiments for this article. KO, MH, SV, RG, and VC conceptualized the work. All authors contributed to the article and approved the submitted version.

## Funding

The Psoriatic Arthritis Program is supported by a grant from the Krembil Foundation. This work was supported by the Krembil Foundation (VC), the Edward Dunlop Foundation (VC) and the Canadian Institutes of Health Research (MDH, #PJT 148565). VC is supported by a Pfizer Chair Research Award, Rheumatology, University of Toronto, Canada. SV was supported for this work by a Young Investigator’s Operating Grant from The Arthritis Society (#TAS-YIO-15-321). AG-A received partial salary support for this work from The Arthritis Society (#TPF-15-123).

## Conflict of Interest

The authors declare that the research was conducted in the absence of any commercial or financial relationships that could be construed as a potential conflict of interest.
