# Corrigendum: Proteinase-Mediated Macrophage Signaling in Psoriatic Arthritis

**DOI:** 10.3389/fimmu.2021.814072

**Published:** 2021-12-15

**Authors:** Fatima Abji, Mozhgan Rasti, Alejandro Gómez-Aristizábal, Carla Muytjens, Mahmoud Saifeddine, Koichiro Mihara, Majid Motahhari, Rajiv Gandhi, Sowmya Viswanathan, Morley D. Hollenberg, Katerina Oikonomopoulou, Vinod Chandran

**Affiliations:** ^1^ Schroeder Arthritis Institute, Krembil Research Institute, University Health Network, Toronto, ON, Canada; ^2^ Department of Physiology & Pharmacology, University of Calgary Cumming School of Medicine, Calgary, AB, Canada; ^3^ Division of Orthopaedic Surgery, Department of Surgery, Toronto Western Hospital, Toronto, ON, Canada; ^4^ Institute of Biomedical Engineering, University of Toronto, Toronto, ON, Canada; ^5^ Division of Hematology, Department of Medicine, University of Toronto, Toronto, ON, Canada; ^6^ Department of Medicine, University of Calgary Cumming School of Medicine, Calgary, AB, Canada; ^7^ Division of Rheumatology, Department of Medicine, University of Toronto, Toronto, ON, Canada; ^8^ Institute of Medical Science, University of Toronto, Toronto, ON, Canada; ^9^ Department of Laboratory Medicine and Pathobiology, University of Toronto, Toronto, ON, Canada; ^10^ Department of Medicine, Memorial University of Newfoundland, St. John’s, NL, Canada

**Keywords:** serine proteinase, spondyloarthritis, monocyte, macrophage, synovial fluid, PAR2, tryptase-6

In the original article, there is a mistake in **Figure 3** as published. Protein concentrations shown in the graph legends are expressed in nM, which were incorrectly shown as nm. The corrected **Figure 3** appears below.

**Figure 3:**
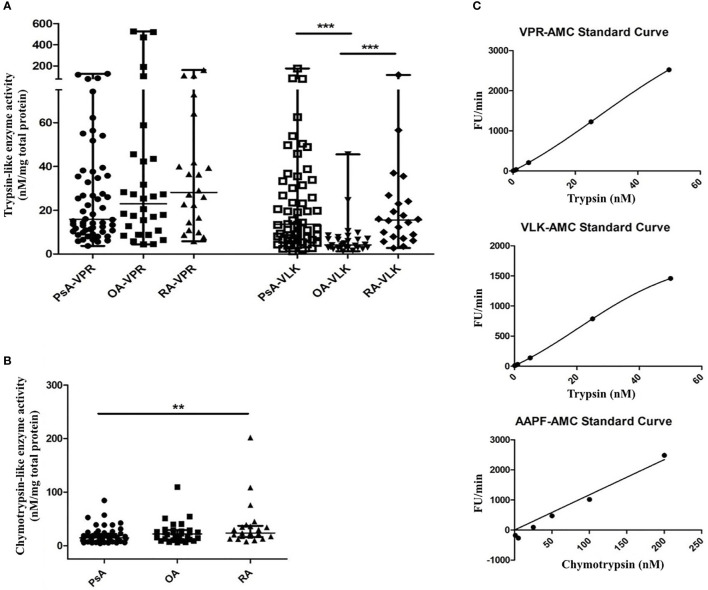
Serine proteinase activity in SF from PsA, RA and OA patients. Serine proteinase activity using fluorogenic substrates for trypsin-like proteinases with a preference for arginine (VPR) or lysine (VLK) **(A)** and chymotrypsin-like proteinases **(B)** is shown. Fluorescence release was monitored for 20 min of a kinetic cycle and the slope of the resulting curve was extrapolated relative to a standard curve of fluorescence release by known concentrations of either trypsin or chymotrypsin and normalized to the total protein concentration of the sample. Representative standard curves are shown in **(C)** for each substrate. Groups were compared using the Kruskal-Wallis test with Dunn’s multiple comparisons test. Asterisks are used to indicate significant differences between groups, where **p ≤ 0.01 and ***p ≤ 0.001.

In the original article, there is a mistake in the legend for **Figure 7** as published. Instead of “5.8 FU/min/mg” it should read “5.8 FU/min/ng”. The correct legend appears below.

In the original article, there is also a mistake in the legend for **Figure 9** as published. Instead of “limits detection”, it should read “limits of detection”. The correct legend appears below.

In the original article, there is a text error. An incorrect instrument source was provided. Instead of “Mandel Scientific”, it should say “BMG Labtech, Ortenberg, Germany”.

A correction has been made to **Materials and Methods, Calcium Cell Signaling Assay, paragraph 1**:

**“**Cultured PAR-responsive human embryonic kidney (HEK)-293 cells were used to establish the ability of SF samples to activate PAR2, as described elsewhere (52). HEK-293 cells were cultured to confluence in DMEM medium containing high glucose and L-glutamine (Millipore Sigma, D5796) supplemented with 10% fetal bovine serum (FBS, Thermo Fisher Scientific, 12484028) and 100 U/ml penicillin/streptomycin (Thermo Fisher Scientific, 15140122). Cells were plated in black 96-well plates at 40,000 cells per well and left to stabilize overnight in a 37°C/5% CO2 incubator. The calcium-regulated fluorescent intracellular calcium indicator, Fluo-4 AM (Thermo Fisher Scientific, F36206) was used to monitor real-time elevations of intracellular calcium following activation or inhibition of PAR2, according to the manufacturer’s instructions. The PAR2 activating peptide, 2-furoyl-LIGRLO-NH2 (2fLI) and the selective PAR-2 inhibitor, I-191 [International Publication No. WO 2015/048245A1 (PCT/US2014/057390)], were used (53–55). Fluorescence was normalized to the signal generated by the calcium ionophore A23187 used at a concentration of 2 µM (Millipore Sigma, C7522). Calcium ionophore was used as a positive control to reflect calcium signaling by Fluo-4-loaded cells, as done previously (56). Data were acquired on a FLUOstar Omega plate reader at 37°C with excitation at 494 nm and emission at 516 nm (BMG Labtech, Ortenberg, Germany).”

In the original article, there is another text error. Instead of nm, the units should be nM.

A correction has been made to **Results**, **Identification of Serine Proteinases in Synovial Fluid, paragraph 1**:

“Investigations of serine proteinase activity in samples from PsA patients have not yet been performed. This unmet need, together with our finding that serine proteinases which signal through PAR2 can be present in PsA SF (22) and combined with the availability of various tools for detecting total serine proteinase activity, led us to focus on this group of proteinases for further study. The presence of serine proteinase activity in SF samples from patients with PsA, RA and OA was confirmed by a fluorogenic proteinase substrate activity assay. A significantly higher level in trypsin-like activity of serine proteinases with a preference for lysine (VLK-AMC substrate) was found in SF from patients with PsA (median 13.51 nM/mg total protein, range 6.58–32.84, p <0.0001) and RA (median 15.57 nM/mg total protein, range 7.73–24.73, p=0.0002) compared to OA (median 4.21 nM/mg total protein, range 2.89–7.28). No significant differences in trypsin-like activity of serine proteinases with a preference for arginine (VPR-AMC substrate) were found between patient groups (**Figure 3A**). Chymotrypsin-like activity was higher in RA (median 23.70 nM/mg total protein, range 16.29–37.03) compared to PsA SF (median 14.55 nM/mg total protein, range 8.59–22.10, p=0.005), with OA SF levels (median 21.93 nM/mg total protein, range 11.40–29.21) not significantly different to either RA (p=0.718) or PsA SF levels (p=0.129; **Figure 3B**). Representative standard curves of active trypsin and chymotrypsin for each substrate are shown in **Figure 3C**.”

In the original article, there is another text error. Instead of “tryptase-2”, it should be “tryptase-6”.

A correction has been made to **Results, Tryptase-6 Triggers Calcium Signals in HEK-293 Cells Partially *via* PAR2, paragraph 1**:

“The next goal was to investigate whether active tryptase-6 could stimulate a calcium signaling response in intact cells and to determine whether this response was mediated by PAR2. Initially the widely-used PAR-responsive cell line, HEK-293, was used. Treatment of HEK-293 cells with the PAR2 peptide agonist (2fLI) caused a concentration-dependent increase in calcium signaling with an EC50 of 0.14 µM (**Figure 6A**). As shown in **Figure 6B**, the PAR2-selective inhibitor, I-191, successfully blocked the PAR2 calcium response when cells were pre-treated with increasing concentrations of I-191, followed by 0.15 µM of 2fLI. The IC50 for the ability of I-191 to block PAR2 signaling was 2.1 nM (**Figure 6B**). Thus, stimulation of wild-type HEK cells with 0.15 µM of 2fLI (**Figure 6C**) was inhibited 99% by 10 nM of I-191 (**Figure 6D**). Treatment of the same wild-type HEK cells with 10.0 FU/min/ng tryptase-6 isolated from the PsA SF by antibody affinity chromatography caused an increase in intracellular calcium (**Figure 6E**) which was inhibited 34% by 10 nM of I-191 (**Figure 6F**). In addition, calcium signaling upon stimulation with tryptase-6 was assessed in CRISPR-eliminated PAR2 knockout HEK-293 cells (**Figure 7**). The PAR2-null cells were not responsive to the potent selective PAR2 peptide agonist, 2fLI (5 µM), but were still sensitive to 2.5 µM of the receptor-selective PAR1 agonist, TFLLR-amide (**Figure 7**, tracing A). In these PAR2-null/PAR1-expressing HEK cells, treatment with 5.8 FU/min/ng of PsA SF-derived tryptase-6 caused an elevation of intracellular calcium that was desensitized to the second challenge with tryptase-6. The tryptase-6-desensitized cells no longer responded to a PAR1-associated response caused by stimulation with 0.5 U/ml of thrombin (open circle, first tracing in **Figure 7B**). In contrast, a robust response to thrombin was observed for the cells not pre-treated with tryptase-6 (right-hand tracing, open circle, **Figure 7B**). Thus, tryptase-6 was able to prevent thrombin’s ability to activate PAR1 either by signal desensitization or by removing the PAR1 tethered ligand so as to “disarm” PAR1. The ability of tryptase-6 to activate MAPKinase signaling was also assessed in HEK-293 cells by western blot analysis. In keeping with the calcium signaling data, western blot detection of activated phospho-MAPKinase caused by tryptase-6 was able to demonstrate the activation of MAPKinase in both PAR2-expressing and PAR2-null HEK cells (data not shown). These results indicate that tryptase-6 stimulates both calcium and MAPKinase signaling in HEK-293 cells not only *via* PAR2 but also *via* a PAR2-independent mechanism. Therefore, in the microenvironment of the SF containing tryptase-6, multiple receptors on monocytes/macrophages or other cell types in principle can be activated by this proteinase, including PAR2.”

**Figure 6:**
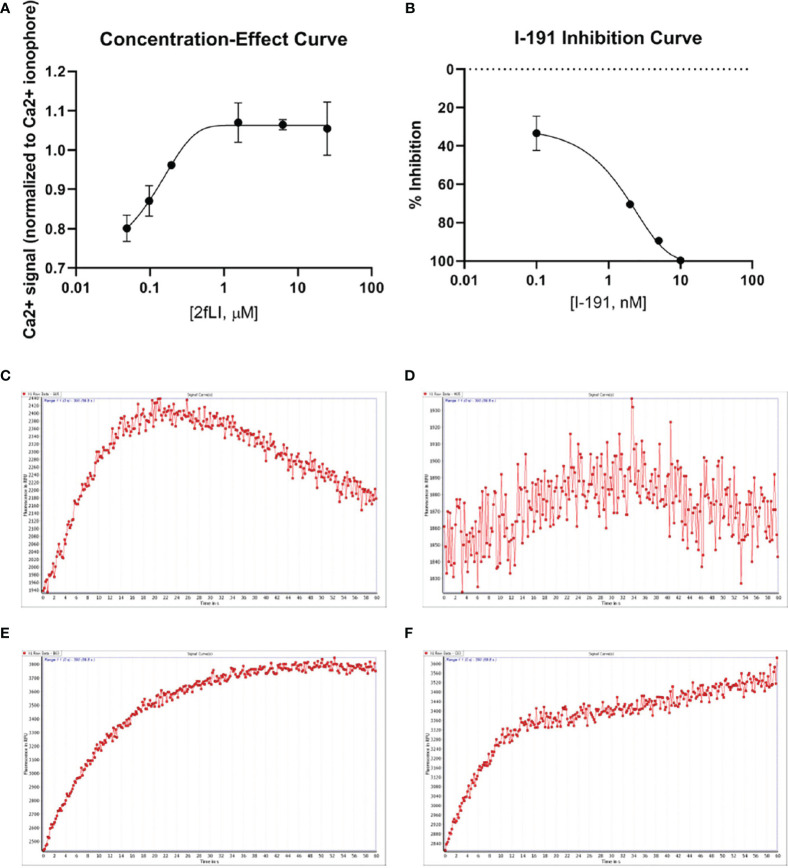
PAR2 and tryptase-6 trigger calcium signals in HEK-293 cells. Calcium signaling assay using the fluorescent indicator, Fluo-4 AM, was used to confirm the signaling potential of PAR2 and determine whether tryptase-6 can elicit a calcium response via PAR2 in HEK-293 cells. The concentration-effect curve for the PAR2 agonist 2fLI with an EC50 of 0.14 µM **(A)** and the concentration–inhibition curve for the PAR2 inhibitor I-191 with an IC50 of 2.1 nM **(B)** are shown. Stimulation of cells with 0.15 µM of 2fLI **(C)** was inhibited 99% by 10 nM of I-191 **(D)**. Stimulation of cells with 10.0 FU/min/ng tryptase-6 isolated by antibody affinity chromatography from PsA SF caused an elevation of intracellular calcium **(E)** and 10 nM of I-191 caused a 34% inhibition of this signal **(F)**. Calcium traces are shown with agonists (2fLI and tryptase-6) added at time zero with or without pre-treatment with I-191 (x-axis=time post addition of agonist, y-axis=units of fluorescence due to calcium release).

**Figure 7:**
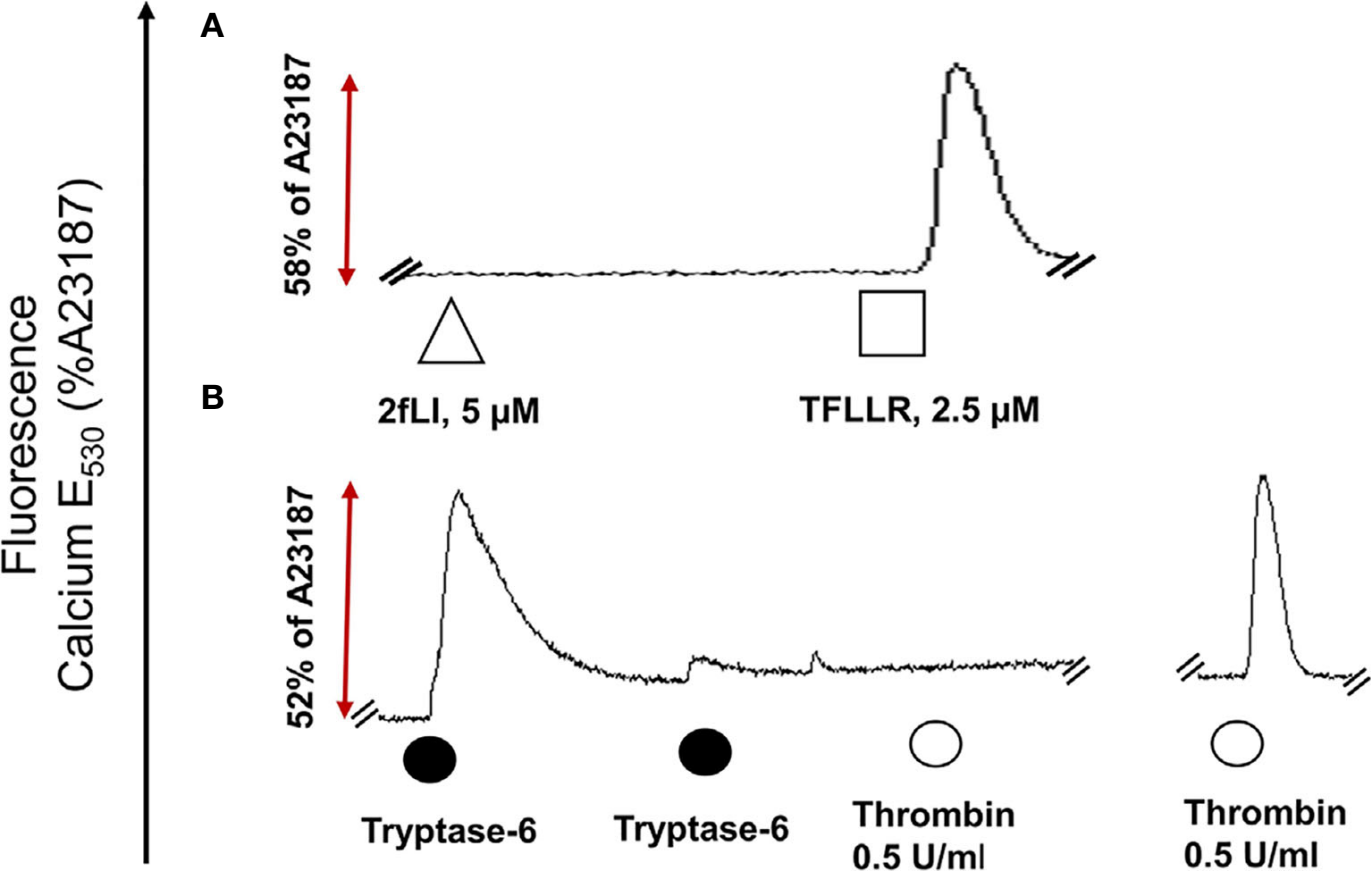
Tryptase-6 triggers calcium signals in PAR2 knockout HEK-293 cells. The calcium signaling assay [fluorescence emission at 530 nm, as a percentage of the signal generated by the calcium ionophore A23187 (%)] was used to determine whether tryptase-6 can trigger calcium signaling *via* PAR2-independent pathways. Tracing **(A)**: The absence of a response to the potent PAR2-selective agonist, 2fLI (open triangle) but presence of a response to the selective PAR1 peptide agonist, TFLLR-amide (open square) confirmed the absence of functional PAR2 and the presence of functional PAR1 in the HEK-293-PAR2-null cells. Tracing **(B)**: Volume equivalent to 5.8 FU/min/ng of PsA SF-derived tryptase-6 (solid circle) elicited a robust calcium response (dark circle), that was desensitized by the second consecutive exposure to tryptase-6 (second solid circle, Tracing **B**). These tryptase-6- treated cells no longer responded to the PAR1 activator, thrombin (0.5 U/ml, open circle, first tracing panel **B**). Cells not treated with tryptase-6 showed a strong calcium signal caused by thrombin (right-hand tracing, panel **B**).

**Figure 9:**
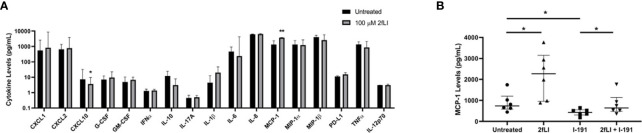
“PAR2 modulates MCP-1 expression from PsA monocytes/macrophages. The expression of 20 chemokines/cytokines measured from conditioned medium of blood-derived PAR2-expressing monocytes/macrophages of PsA patients (n=8) using a multiplex Luminex assay is shown in **(A)**. Cells were cultured for 48 h in the presence or absence of 100 µM 2fLI and stimulated with LPS during the last 4 h. The expression of IFNg, IL-17E and IL-4 were below the limits of detection and are not shown. Values are graphed on a log10 scale to better visualize differences between groups. MCP-1 was also measured in additional samples of blood-derived PAR2-expressing monocytes/macrophages (n=6) in the presence of 30 µM I-191, 100 µM 2fLI or both **(B)**. Expression was compared between groups using the Wilcoxon matched-pairs signed rank test. Error bars indicate the median ± interquartile range. Asterisks are used to indicate significant differences between groups, where *p < 0.05 and **p < 0.01”.

The authors apologize for these errors and state that these do not change the scientific conclusions of the article in any way. The original article has been updated.

## Publisher’s Note

All claims expressed in this article are solely those of the authors and do not necessarily represent those of their affiliated organizations, or those of the publisher, the editors and the reviewers. Any product that may be evaluated in this article, or claim that may be made by its manufacturer, is not guaranteed or endorsed by the publisher.

